# Selecting and Using the Appropriate Influenza Vaccine for Each Individual

**DOI:** 10.3390/v13060971

**Published:** 2021-05-24

**Authors:** Toshiki Sekiya, Marumi Ohno, Naoki Nomura, Chimuka Handabile, Masashi Shingai, David C. Jackson, Lorena E. Brown, Hiroshi Kida

**Affiliations:** 1 International Institute for Zoonosis Control, Hokkaido University, Kita-20 Nishi-10, Kita-ku, Sapporo 001-0020, Japan; tsekiya@czc.hokudai.ac.jp (T.S.); ohnom@czc.hokudai.ac.jp (M.O.); nomura@czc.hokudai.ac.jp (N.N.); chimuka94@czc.hokudai.ac.jp (C.H.); shingaim@czc.hokudai.ac.jp (M.S.); 2 International Collaboration Unit, International Institute for Zoonosis Control, Hokkaido University, Sapporo 001-0020, Japan; davidcj@unimelb.edu.au (D.C.J.); lorena@unimelb.edu.au (L.E.B.); 3 The Department of Microbiology and Immunology, The University of Melbourne at the Peter Doherty Institute for Infection and Immunity, Melbourne 3000, Australia; 4 Collaborating Research Center for the Control of Infectious Diseases, Nagasaki University, Nagasaki 852-8521, Japan

**Keywords:** seasonal influenza vaccine, whole virus particle vaccine, pandemic preparedness, priming immune response

## Abstract

Despite seasonal influenza vaccines having been routinely used for many decades, influenza A virus continues to pose a global threat to humans, causing high morbidity and mortality each year. The effectiveness of the vaccine is largely dependent on how well matched the vaccine strains are with the circulating influenza virus strains. Furthermore, low vaccine efficacy in naïve populations such as young children, or in the elderly, who possess weakened immune systems, indicates that influenza vaccines need to be more personalized to provide broader community protection. Advances in both vaccine technologies and our understanding of influenza virus infection and immunity have led to the design of a variety of alternate vaccine strategies to extend population protection against influenza, some of which are now in use. In this review, we summarize the progress in the field of influenza vaccines, including the advantages and disadvantages of different strategies, and discuss future prospects. We also highlight some of the challenges to be faced in the ongoing effort to control influenza through vaccination.

## 1. Introduction

Influenza A virus poses a worldwide and ongoing threat to human health, causing 290,000–600,000 deaths and up to 5 million cases of severe illnesses annually [1]. This is accompanied by a significant negative impact on the global economy, in part due to the cost of medical care and loss of productivity. In the United States of America alone, the economic impact attributed to seasonal influenza is estimated at USD 6.3~25.3 billion a year [2]. Moreover, in the case of an outbreak of pandemic influenza, the impact can be much more severe, as was experienced in 2009.

Vaccination is the most cost-effective way to combat influenza. Vaccines, which primarily induce neutralizing antibodies against the viral surface glycoproteins, hemagglutinin (HA) and neuraminidase (NA), have been available since the 1940s and have greatly reduced the incidence of disease following infection and saved numerous lives [3,4,5]. Currently three types of influenza vaccine, “inactivated”, cold-adapted “live attenuated”, and “recombinant HA” vaccines, are licensed for human use in different countries [3] (Table 1). These vaccines are usually available as trivalent or quadrivalent formations, containing recent influenza A virus strains of the H1N1 and H3N2 subtypes in combination with one or two influenza B virus strains of the Yamagata and/or Victoria lineages. Each type of vaccine has its own advantages and disadvantages (summarized in Table 2), so users need to understand their characteristics and choose the appropriate vaccine.

During 2018 and 2019, the Centers for Disease Control and Prevention estimated that influenza vaccination prevented approximately 4.4 million illnesses, 2.3 million medically attended illnesses, 58,000 hospitalizations, and 3500 deaths associated with influenza in the United States [6]. Clearly immunization against influenza is highly beneficial and is a global imperative. However, some problems still remain with influenza vaccines, a major one being the reduced effectiveness of the most commonly used influenza vaccines in the elderly and young children [5]. Therefore, it is desirable to have vaccines with high antigenicity that are able to protect immunodeficient individuals, such as the elderly, and to prime naïve individuals such as young children. The other problem is that their effectiveness is largely dependent on how well matched the vaccine strains are with those circulating in the upcoming influenza season. As a result of continual viral antigenic drift and/or shift, the vaccine effectiveness wanes over time and vaccine strains need to be regularly updated to maintain protection of the target population. For this reason, strains of influenza virus in the vaccine are chosen annually by the World Health Organization, based on the global surveillance of circulating strains. Unlike current vaccines, ones that could induce cross-protective immunity would be of major benefit in preventing influenza, even if the vaccine strains are a poor match for the newly circulating strain.

In this review, we summarize current influenza vaccines and discuss some of the advantages and disadvantages associated with these. We also discuss progress in the development of new influenza vaccines and the distinctive advantages they are expected to provide. Currently, in many countries, only limited types of influenza vaccine are approved or commercially available. It is hoped that in the future a broader spectrum of influenza vaccines will become widely available so that individuals can receive the vaccine that best suits their needs.

## 2. Currently Licensed Influenza Vaccines

### 2.1. Inactivated Vaccines

There are three types of inactivated vaccines: whole virus particle vaccine (WPV), split virus vaccine (SV), and subunit vaccine. WPV is prepared by propagation of virus in embryonated chicken eggs and purification of virus particles followed by chemical inactivation with formaldehyde and/or β-propiolactone. SV is prepared by disruption of the purified influenza virus by ether and/or detergent. Subunit vaccines are highly enriched with HA and NA by adding an extra purification step to exclude other viral components, such as the viral nucleoprotein (NP), genomic RNA, matrix protein (M1) and viral envelope. Production of the vaccine strains in embryonated chicken eggs provides high yields at relatively low cost. However, the necessary egg-adaptation of vaccine seed strains to increase the virus yield for mass production may sometimes introduce unwanted egg-adapted mutations, such as the HA substitutions L194P [7,8] in H3N2, and Q226R [9] in H1N1, which have been shown to significantly affect vaccine effectiveness. Furthermore, non-viral contaminants such as egg proteins need to be removed. To avoid such unwanted mutations and contaminants occurring during virus propagation in eggs, mammalian cell culture based subunit vaccines, such as Flucelvax^®^, are licensed in many countries [10]. Unlike embryonated chicken eggs, the use of cell culture obviates the egg-adaptation process of vaccine strains, allowing rapid scaling up of production. However, the high production cost and low vaccine yield provide a challenge for this cell culture technology in competing with the embryonated egg platform.

#### 2.1.1. Whole Virus Particle Vaccine (WPV)

An inactivated whole influenza virus particle vaccine consisting of purified virus inactivated with formalin was first licensed in the United States in the 1940s [11]. Despite the higher immunogenicity exhibited in animals and humans, it was subsequently abandoned due to adverse effects, in particular the induction of symptoms typical of an excessive inflammatory response, such as fever, redness, and swelling at the injection site. This vaccine was replaced by the less “reactogenic”, inactivated SV [12,13]. Although the reduced reactogenicity of the SV is an advantage, the requirements for larger amounts of antigen and the addition of an adjuvant to compensate for the low immunogenicity are seen as disadvantages.

In recent years, a variety of different forms of influenza vaccines have been investigated in an attempt to improve their ability to protect humans against influenza, and a number of studies have re-evaluated the use of WPV as a vaccine. The foundation for this follows reports that WPVs are more immunogenic than SVs or subunit vaccines, particularly in immunologically naïve humans and animals [14,15,16,17,18,19]. Importantly, the concerns about the high reactogenicity that have been reported to be associated with the use of WPVs are thought to be, in part, the result of residual egg-derived products carried through from the production process [20], which can now be lessened through the implementation of better purification techniques and/or the use of cell culture-based production systems [21,22,23]. Several clinical trials have since reported a similar degree of reactogenicity of WPV to that of SVs [24,25,26,27,28,29], and some of these studies also reported higher seroconversion rates using lower doses of WPV compared to SV [24,25,28]. Furthermore, additional “dose-sparing” effects of WPV can be achieved in small animal models without [17], or with, adjuvants such as CoVaccine HT [30] or cationic lipid/DNA complexes [31]. These dose-sparing effects are thought to be due to (1) the particulate nature of WPV virions, which possess a higher density of antigenic proteins compared to detergent disrupted soluble antigens that make up a SV [16], and/or (2) the presence of viral RNA in WPV, which can signal through Toll-like receptor (TLR) 7 to potentially provide an adjuvant effect [32,33]. Although SV also contains RNA, it is not efficiently endocytosed to trigger the TLRs in the endosome (manuscript in preparation). Moreover, the potential of WPV to induce cross-protective responses has been reported [34,35,36]. For emerging pandemic influenza viruses, where most of the population is immunologically naïve to these antigenically novel viruses, WPVs with or without an adjuvant have been tested and licensed as pre-pandemic vaccines [29,37]. Thus, the advantage of WPV is its strong immunogenicity and ability to prime naïve children [38], while the disadvantage is the concern of fever.

#### 2.1.2. Split Virus Vaccine (SV) and Subunit Vaccine

The most commonly used vaccine against seasonal influenza is ether- or detergent-disrupted SV. SVs are proven to induce moderate immune responses in previously infected or vaccinated individuals with minimum adverse effects but are less immunogenic in the young and elderly populations [5]. For this reason, in vaccines for the elderly population in particular, the amount of HA antigen is increased to compensate for a weakened immune response. On the other hand, it is recommended that young individuals, between 6 months and 8 years old, receive two inoculations to achieve sufficient immunity [3]. The highly specific nature of the immunity induced by these vaccines requires their frequent update and administration to ensure the relevance of the component virus strains.

Subunit vaccines are the most purified vaccine within the licensed inactivated influenza vaccines, as an extra purification step is taken to separate HA and NA from other viral components, as well as egg contaminants, after splitting the virus. However, because of the lack of immunostimulants in these vaccines, the efficacy of the vaccine is also quite low. Therefore, subunit vaccines for the elderly population include an adjuvant (Fluad^®^) to improve the effectiveness of the vaccine [39,40].

Despite advances in manufacturing facilities and technologies, the timeline for the seasonal influenza vaccine, especially SV and subunit vaccine production, from strain selection to distribution of a vaccine has not hugely changed and takes around 6 months before being available to the public [41]. The addition of extra purification steps and splitting virus in SV and subunit vaccines takes longer than other seasonal influenza vaccines, such as WPV or live attenuated vaccine. This is exceedingly problematic when choosing virus strains that are likely to become predominant in circulation, leaving little opportunity to change the vaccine components if they are mismatched to the emerging strains. Even more challenging is the case of pandemic strain emergence, when a vaccine is required quickly for the entire population.

Both SV and subunit vaccines have the advantage of less adverse reactions, such as fever, compared to WPV, but the weak immunogenicity and lack of priming ability are disadvantages.

### 2.2. Live Attenuated Vaccine

The cold-adapted live attenuated influenza vaccine contains strains of influenza virus that have been derived by reassortment with a cold-adapted influenza virus that replicates only at temperatures lower than those that prevail in the lower respiratory tract [42,43]. The attenuated virus in the vaccine, when administered intranasally, replicates to a limited extent in the upper respiratory tract. The advantages of this vaccine over the SVs described above is that it mimics a natural infection without causing serious influenza illness and induces antibodies of both IgA, which is an important isotype for mucosal immunity to provide initial protection against influenza viruses at the entry site, and IgG, which is the most important class of antibody for neutralization of influenza viruses in the tissues and blood stream [44]. Furthermore, live attenuated influenza vaccine is reported to give better cellular immunity, including T cell responses that are cross-reactive against heterologous influenza virus strains [45,46,47]. In addition, live attenuated influenza vaccines have been shown to give a better protection in young children than inactivated influenza vaccines [47,48,49]. The disadvantage of live attenuated influenza vaccines, however, is that they are not recommended for use in children younger than 2 years of age, people older than 18 years (Europe), or 50 years (United States) of age, pregnant women, or immunocompromised individuals, as the viruses in the vaccine may show a greater replication in those with suboptimal immune systems, to the point of harm rather than benefit [50,51]. Likewise, family members or household contacts should not receive live vaccines for fear of transmission to immunodeficient individuals [52]. There is also concern about the emergence of pathogenic viruses due to genetic reassortment with a coinfecting influenza virus and also reversion to virulence mutations [53]. Therefore, the advantage of live attenuated vaccines is their ability to induce both IgA and IgG antibodies and a cellular immunity similar to that induced by natural infection, while the disadvantages are the unsuitability for certain vulnerable target groups within the population and the risk of generating pathogenic viruses.

### 2.3. Recombinant HA Vaccine

The recombinant HA vaccine is produced by a recombinant protein expressing system using insect cells and baculovirus. The first such vaccine was approved by the Food and Drug Administration for use in the United States as Flublok^®^ (Trivalent) in 2013, which was subsequently replaced by a quadravalent version in 2017 and more recently approved by the European Commission to use in Europe as Supemtek^®^ in 2020. Since this platform does not rely on live virus adaptation and propagation in embryonated eggs, unwanted mutations in the HA gene do not arise. Moreover, the manufacturing process of recombinant HA vaccines can be completed within 2 months, indicating that this vaccine potentially allows a quick response to emerging situations, such as pandemics [54]. Like other licensed vaccines, annual updating of the HA sequence used is necessary to match with those of the predicted circulating strains. Furthermore, licensed recombinant HA vaccines contain three times the amount of HA compared to typical SVs to induce sufficient HA-specific antibody responses in the absence of immunostimulants. Furthermore, the fact that these vaccines contain no NA, unlike inactivated and live attenuated influenza vaccines, means they lack an additional mechanism for suppressing infection, as antibodies against NA can enhance protection [55,56]. Nonetheless, a recent study by Richards et al. [57] showed that recombinant HA vaccine induced better HA-specific CD4^+^ T cells compared to egg-derived SV (Fluzone^®^) or cell-derived subunit vaccine (Flucelvax^®^) in vaccinated humans, which they speculated could be due to the greater amount of HA in the vaccine, a lack of competition with T cells to other virus components, or the different glycosylation pattern of insect cell-derived HA, which could alter the antigen handling and immunogenicity. The usage of this vaccine is recommended for people 18 years old or older and who are thought to be already primed. Thus, the advantages are the shorter production time, due to no adaptation process to eggs and no unwanted mutations of HA protein, while the disadvantages are age restriction and requirement for three times more antigen.

## 3. Recent Progress of Influenza Vaccine Research

Over the last few decades, our understanding of the immune system and the various states of immunity, as well as the development of new techniques in microbiology and immunology, has grown and, as a consequence, vaccine design is now moving from the empirical approaches that use attenuated or inactivated whole pathogens to more refined approaches that focus on inducing an appropriate and safe immune response using specified antigenic targets with efficient and safe modes of vaccine delivery. Some of the vaccine technologies that have been explored for influenza are discussed below.

### 3.1. DNA-Based Vaccines

DNA-based vaccines against influenza virus have been actively investigated since the 1990s. Unlike traditional influenza vaccines, this technology does not require amplification of live viruses, as the target viral protein (mainly HA) is produced directly by host cells that have taken up the injected DNA vaccine. DNA vaccines can be quickly upscaled to produce large quantities, which is important in urgent cases such as emerging pandemic influenza. Moreover, production does not require high-level biocontainment facilities, which is an advantage for producing highly pathogenic avian influenza or pandemic influenza vaccines. There have been a number of reports that DNA-based vaccines can efficiently induce both humoral and cell-mediated immune responses in a mouse models [58], but despite the success in small animals, the induction of effective immune responses in larger animal models, such as in macaques, has been difficult to reproduce [58]. The theoretical risk of integration of the vaccine DNA sequences into the host genome has also raised concerns about the development of these vaccines for human use. Nonetheless, improvements in DNA vaccine delivery systems, such as the use of microneedles [59,60], the Biojector system [61], or electroporation [62], have been found to induce greater humoral and adaptive immune responses compared to traditional intramuscular administration. Several DNA vaccine candidates have now entered Phase I clinical trials and have shown some benefits for priming immune responses against seasonal and pandemic viruses [63,64,65].

### 3.2. mRNA-Based Vaccines

The recent outbreak of COVID-19, has opened the door for mRNA vaccines to obtain approval for use in humans for the first time [66]. Prior to this, mRNA vaccines had not been considered actively for human use due to concerns regarding their stability above ultra-low temperatures: mRNA vaccines need to be stored at −80 °C, making it extremely hard to sustain a cold-chain. However, the enormous global toll of COVID-19, provided the impetus for developing infrastructure to maintain a cold-chain to deliver desperately needed vaccines against the SARS-CoV-2 virus. Having this infrastructure in place may make it easier for pharmaceutical companies to develop mRNA vaccines for other diseases.

There are two main types of mRNA vaccines that have been developed against influenza virus: the non-replicating mRNA vaccines and the self-amplifying mRNA vaccines [67]. Both approaches are currently being extensively investigated by major pharmaceutical companies, such as Moderna Therapeutics (Cambridge, MA, USA) [68] and CureVac AG (Tübingen, Germany). Moderna Therapeutics is developing a non-replicating mRNA vaccine with modified nucleosides that is incorporated into lipid nanoparticles (LNP) and is planning to begin a phase I clinical trial for seasonal influenza vaccine in 2021 [69]. CureVac AG, on the other hand, chose a strategy based on sequence-optimized unmodified mRNA–LNP [70]. Although, self-amplifying mRNA vaccines for influenza virus have not entered into clinical trial, there has been progress in the development of these vaccines for COVID-19 [71]. One advantage of mRNA-based vaccines, as illustrated by those used in the COVID-19 pandemic, is that they can be quickly produced once the genomic sequence of the immunological target has been identified. As the surveillance and identification of novel HA sequences arising by antigenic drift is routine for influenza viruses, mRNA vaccines may well have the advantage of being the fastest of the influenza vaccines to be produced and accessible to the public. Therefore, we may well see mRNA-based vaccines for influenza in the near future.

### 3.3. Vector-Based Vaccines

Another vaccine platform that has been highlighted by the outbreak of COVID-19 is vector-based vaccines. Although vector-based vaccines are widely used in veterinary medicine, human licensed vector-based vaccines had been limited to use against Ebolavirus [72] until the recent approval of AstraZeneca’s chimpanzee adenovirus-based (ChAdOx1) nCoV-19 vaccine against SARS-CoV-2 (Vaxzevria^®^) [73].

The principle of vector-based vaccines is delivering target antigens encoded in attenuated or replication-defective viruses that can target specific host cells, and then antigens are expressed to induce antigen-specific immune responses. As the vectors can effectively activate innate immune responses through TLR dependent and independent pathways to induce a strong immune response, the vector itself acts as the adjuvant [74]. For influenza vaccine, a number of different type of vectors have been utilized and investigated in clinical trials and non-human primate animal models including: adenovirus [75,76,77,78], recombinant vaccinia virus [79], and Newcastle disease virus [80] for enhancing neutralizing antibody against HA; chimpanzee adenovirus [81,82] for inducing T cell response for conserved internal proteins, such as NP and M1; and modified vaccinia virus Ankara (MVA) [82,83,84,85,86], used as both an antibody and T cell targeting vaccine. An effective T cell response against conserved internal proteins of influenza virus may be important for cross-reactive immune responses (discussed more detail in Section 3.4).

Although the induction of strong immune responses has been reported in clinical trials, concerns remain about the use of vectors based on common human pathogens, such as adenovirus, because of the potential for the vector-based vaccine to be neutralized by pre-existing immunity against the vector before immunity to the target antigen is induced [87]. To avoid these concerns, the use of non-human virus vectors such as chimpanzee adenovirus [81] or the use of two different virus vectors to prime and boost immune response has been examined [82].

It remains unclear how extensive use of Vaxzevria^®^ in a wide population affects the efficacy of the next vector-based vaccine that uses the same vector platform; vector-based vaccines may play some role in influenza vaccines.

### 3.4. Universal Vaccines

One factor that limits the efficacy of existing SV and subunit vaccines is their inability to induce effective cell-mediated responses, especially those mediated by CD8^+^ T cells. The benefit of these responses, particularly if directed against internal viral proteins, such as the NP [88] and the M1 [89], is that they are cross-reactive, recognizing a wide range of influenza A strains. Although influenza-specific cytotoxic CD8^+^ T cells cannot prevent infection, as they only act upon virus-infected cells, the presence of these cells has been shown to be associated with better prognosis following influenza virus infection. As such, vaccines with the capacity to establish a pool of memory cross-reactive CD8^+^ T cells have the potential to be “universal” vaccines, that is those providing broad coverage against heterologous influenza A virus strains, including those of different subtypes [90,91,92]. The importance of CD8^+^ T cell responses in resolving influenza virus infection is illustrated in a study of zoonotic H7N9 infections in humans, where Wang et al. [93] found that the presence of an early CD8^+^ T cell response, indicative of activation and expansion of pre-existing cross-protective memory cells, correlated with faster recovery and less severe disease. In fact, many experimental influenza vaccines based on protein-subunits [36,94], peptide epitopes [95,96,97], virus-like particles (VLPs) [98], or DNA [99,100] have demonstrated that the induction of CD8^+^ T cell responses cross-protects against heterologous viral challenge in small animal models. Those based on protein-subunits and peptides in particular, require a delivery system that provides efficient entry into dendritic cells for T cell priming. It is also necessary that sufficient CD8^+^ T cell epitopes are identified and are present in these vaccines, in order to provide broad coverage of the diverse HLA haplotypes of the target population. Once a CD8^+^ T cell response has been primed, the cells enter a memory phase and the time taken to reactivate these upon subsequent infection and to expand the cell population to sufficient numbers to effectively clear infected cells is affected by various factors, including the waning of immunity [101,102]. Nonetheless, these responses play an important role in significantly reducing viral load and ameliorating disease severity [103].

The alternative approach, of inducing cross-reactive antibodies to conserved epitopes, particularly those on the HA stalk, has also been actively investigated for a universal vaccine against influenza virus [104,105,106], but the form of the HA required to induce these normally subdominant antibodies has been challenging. Stalk antibodies do not function as classical neutralizing antibodies, but have been proposed to work by inhibiting virus release from the endosome or by inhibiting cleavage of the HA precursor HA0 to its mature form. The fact that stalk specific antibodies require Fc-mediated interactions to function in vivo suggests that antibody dependent cell-mediated cytotoxicity (ADCC) and complement-dependent cytotoxicity of infected cells are the major mechanisms of action [107]. Cross-reactive, stalk-specific antibodies have not shown any great impact on disease severity in animals, and the results of human studies to date do not support any firm correlation of stalk antibodies with protection [108].

## 4. Revisited Whole Virus Particle Vaccine in Super-Pure Form

Increasing interest in the use of WPV as a seasonal influenza vaccine due to its ability to prime naïve children led us to strive to bring WPV back to the market in Japan. To achieve this goal, we established the All-Japan Influenza Vaccine Study Group, which includes all four Japanese seasonal influenza vaccine manufacturers. WPV has been produced by the four manufacturers from the same batch of vaccine used for their SV preparations. The egg-derived vaccine viruses are highly purified using state-of-the-art technologies. When the immunogenicity of the WPVs were examined alongside the corresponding SVs in naïve mouse and macaque models [17], the results showed greater levels of hemagglutination-inhibiting antibodies, which led to protection from lethal homologous influenza virus challenge. These results with WPV were achieved using only one fifth of the HA dose of SV, an indication of substantial dose sparing. Moreover, adverse effects, such as fever, were within a comparable range to those seen with SVs in macaques (manuscript in preparation). The ability to compare WPV with the corresponding SV made by each different vaccine manufacturer provided a unique opportunity, and the superiority of WPV over SV seen with each paired comparison has prompted us to investigate further the candidate WPVs in the macaque model and to progress to Phase I and II clinical trials. Therefore, this new type of WPV has the advantages of strong immunogenicity, the ability to prime naïve children, and less adverse reactions derived from egg contaminants. As an improved version of an already approved and effective vaccine, super purified WPV seems to be a very realistic candidate for a new seasonal influenza vaccine.

## 5. Adjuvants

One potential approach to improving the efficiency of influenza vaccines is the use of adjuvants. In fact, there are adjuvants already licensed for use in human influenza virus vaccines including Alum, MF59, and AS03 (against pandemic influenza) [109]. Benefits of adjuvants differ according to the type used in the vaccine but generally show one or more of the following advantages:

### 5.1. Enhancing Immunogenicity of Antigens

Antigens in the vaccine, especially in subunit vaccines, are poor at inducing immune responses due to a lack of immunostimulatory components, such as pathogen-associated molecular patterns (PAMPs), which are often present in the intact pathogen. The absence of immunostimulants makes it difficult for the immune system to recognize the vaccine as “foreign” and mount effective immunity in response. Receptors recognizing PAMPs include the TLRs, and agonists of these, such as Pam_2_Cys (TLR2), flagellin (TLR5), ssRNA (TLR7/8), or CpG (TLR9), have been used to enhance the immunogenicity of protein antigens such as SV [110], M2e [111], recombinant HA [112], and NP [113], respectively.

### 5.2. Improving the Quality and Type of Responses

Depending on the adjuvant used, the response can be modulated in a controllable manner, for example, by the different cytokines and chemokines that are produced, which can in turn shape the balance of different effectors in the subsequent adaptive immune response [114]. For example, Th1-type cytokines promote a CD8^+^ T cell response, which may be an additional benefit in the fight against influenza virus infection, as discussed in the subtopic, universal vaccines. Th1-type cytokines amplify the antibody response and have anti-inflammatory mediators. Ideally, a balanced response is required and this can be aided by the inclusion of appropriate adjuvants. Additionally, adjuvants such as TLR2 agonists, as they engage with receptors on the cell surface, may also provide a means of entry to the cell for electrostatically- or physically-associated antigens, a requirement for the effective priming of T cells [110,115,116].

### 5.3. Overcoming Immunodeficiency

Many sectors of the population have suboptimally functioning immune systems. These include HIV patients, those on immunosuppressive treatments, and even those at the extremes of age; the very young, whose immune system is not fully developed, and the elderly, whose capacity to respond is waning. These populations do not respond well to traditional SVs but are at the highest risk from influenza. Licensed vaccines, such as Fluad^®^ (containing MF59, the oil squalene as an oil in water preparation) or 3Fluart^®^ (aluminium phosphate), are already available on the market to help induce better immunogenicity in the elderly. Furthermore, AS03, an adjuvant system containing the surfactant polysorbate 80 and two biodegradable oils, [117] increases vaccine efficacy and is licensed for prepandemic influenza virus H5N1 vaccine [118] and H1N1pdm09 vaccine [119].

### 5.4. Dose-Sparing Response

The ability to use lower doses of vaccine antigen to achieve the same level of immunity is most beneficial in a pandemic situation, where manufacturers need to distribute vaccine to the wider population quickly. Dose-sparing also lowers the manufacturing cost for seasonal influenza vaccines.

While the number of adjuvants used for influenza virus vaccine is currently limited, the mechanism of action of these adjuvants is well understood. It is hoped that in the future, additional well characterized adjuvants, such as TLR agonists, can be explored to create vaccines that provide even greater benefits to those individuals whose immune system is not fully functional.

## 6. Conclusions and Future Perspectives

Although the efficacy of a seasonal influenza vaccine heavily depends on how well it matches the circulating influenza viruses, current SV and its egg-based production platform function well for the large portion of the population who have been exposed to influenza virus or vaccinated with SV previously. This, however, could be the reason that seasonal influenza vaccines have not greatly evolved over the last 40 years, despite their low efficacy for certain sectors of the population, including young children. On the other hand, we must acknowledge the effort made by vaccine manufactures to develop the infrastructure for the production of the current seasonal influenza vaccine, which has the potential to produce 1.48 billion seasonal and 8.31 billion pandemic doses globally [120]. Nonetheless, considering the nature of the influenza virus and the increasing demands to consider individual needs, more options for different influenza vaccines should be available. Japan, for example, has only licensed seasonal influenza vaccines based on SV; whereas, live attenuated and recombinant HA vaccines are licensed in other countries. This strategy may leave minor populations at greater risk.

The vaccine response to the COVID-19 pandemic has laid the groundwork for mRNA vaccines, and we may see the rapid development and licensing of these for influenza virus in the near future. These and other rapidly produced influenza vaccines, such as recombinant HA vaccines, would be a tremendous global aid when a vaccine is required in a short time frame due to the inevitable outbreak of an influenza pandemic. Although those vaccines certainly give some degree of protection [121] against infection and help to reduce the severity, as well as spread, of the disease at the beginning, limitations in manufacturing capacity may need to be covered by SV and other large productive capacity platforms. For enhancing the immune response in naïve populations, such as the young, WPV, the original vaccine against influenza virus, is an ideal candidate, as it likely induces vaccine-specific primary immune responses. For the elderly population, the inclusion of adjuvants in the vaccine will help induce better immune responses, and this approach will certainly be developed further as new adjuvants are tested extensively for safety in humans. Altogether, providing for and covering each vaccine’s strong points and weaknesses to protect broader communities will ultimately improve the overall protection against influenza virus infection.

## Figures and Tables

**Table 1:** Currently licensed influenza vaccines in Europe, Japan and United states.

Region	Vaccine Platform	Vaccine Type	Vaccine Name	Manufacturer	Adjuvant	Produced in
Europe	Inactivated	Whole particle virus	3Fluart	Fluart Innovative Vaccines Kft	Alum	Egg
			Afluria	Pfizer/Seqirus	None	Egg
			Influvac Xanaflu	Mylan Products Ltd.	None	Egg
			Influvac Tetra	Mylan Products Ltd.	None	Egg
			Agrippal	Seqirus	None	Egg
		Split virus	Afluria	Pfizer/Seqirus	None	Egg
			Fluarix	GlaxoSmithKline	None	Egg
			Fluarix Tetra	GlaxoSmithKline	None	Egg
			Trivalent Influenza Vaccine High Dose	Sanofi Pasteur	None	Egg
			Vaxigrip Tetra	Sanofi Pasteur	None	Egg
			Vaxigrip Istivac Mutagrip	Sanofi Pasteur	None	Egg
		Subunit	Fluad	Seqirus	Squalene (MF59)	Egg
			Agrippal	Seqirus	None	Egg
			Xanaflu	Abbot Biologicals/Mylan Products Ltd. (Marketing Authorisation Holder)	None	Egg
			Flucelvax Tetra	Seqirus	None	Cell
			Imuvac	Abbot Biologicals/Mylan Products Ltd. (Marketing Authorisation Holder)	None	Egg
	Live attenuated		Fluenz Tetra	AstraZeneca	None	Egg
	Recombinant HA		Supemtek	Sanofi Pasteur	None	Cell
Japan	Inactivated	Split virus	Influenza HA Vaccine“SEIKEN”	Denka Co., Ltd.	None	Egg
			Influenza HA Vaccine“DAIICHI SANKYO”	Daiichi Sankyo Co., Ltd.	None	Egg
			Influenza HA Vaccine“KMB”	KM Biologics Co., Ltd.	None	Egg
			Influenza HA Vaccine“BIKEN”	BIKEN Co., Ltd.	None	Egg
			Flubik HA (Thiomersal-free)	BIKEN Co., Ltd.	None	Egg
United State	Inactivated	Whole particle virus	Afluria	Seqirus	None	Egg
		Split virus	Fluarix	GSK	None	Egg
			FluLavel	GSK	None	Egg
			Fluzone	Sanofi Pasteur	None	Egg
		Subunit	Fluad	Seqirus	Squalene (MF59)	Egg
			Flucelvax	Seqirus	None	Cell
	Live attenuated		FluMist Quadrivalent	AstraZeneca	None	Egg
	Recombinant HA		Flublok	Sanofi Pasteur	None	Cell

**Table 2:** Each Vaccine platform’s advantages and disadvantages.

	Inactivated								
	Whole Virus Particle	Split Virus	Subunit	Live Attenuated	Recombinant HA	DNA and RNA	Vector	Adjuvanted (Oil)	Adjuvanted (TLR Agonist)
Manufacturing Speed	Slow	Slow	Slow	Slow	Fast	Fast	Medium	Slow	Case-by-case
Manufacturing capacity	Large	Large	Small—Medium	Medium	Small	Medium	Medium—Large	Small—Medium	Small
Cost	Low	Low	Low—Moderate	Low	Moderate—High	Moderate—High	Moderate—High	Moderate	Moderate—High
Single dose	Yes	No	No	Yes	No	No	Yes	Yes	Yes
Antibody response	Strong	Weak—Moderate	Weak	Strong	Weak	Moderate	Strong	Moderate—Strong	Strong
CTL response	Yes	No	No	Yes	No	Yes	Yes	No	Yes
Priming Ability	Yes	No	No	Yes	No	Yes	Yes	Yes	Yes
Young	Yes	No	No	Yes	No	Not Available	Not Available	Yes	Not Available
Elderly	Yes	No (except high dose)	No	Not Available	No	Yes	Yes	Yes	Yes
Human License (Influenza vaccine)	Yes	Yes	Yes	Yes	Yes	No	No	Yes	No
Advantage	Dose-sparing effect	Low adverse effect	Low adverse effect Non egg-derived virus	Mimic natural infection Induce IgA Ab	No mutation Non egg-derived virus	No mutation	Mimic natural infection	Give stronger immunity to elderly	Can target more specific immune responses
Disadvantage	May have trace of egg protein	May have trace of egg protein	May have trace of egg protein	Virulence might reverse Cannot use under 2 yrs old	Require 3 times amount of HA protein	Need to store at low temperature	Pre-existing immunity against vector May not work as booster shot		May induce high adverse effect
